# Tailoring Plasmonic Enhanced Upconversion in Single NaYF4:Yb^3+^/Er^3+^ Nanocrystals

**DOI:** 10.1038/srep10196

**Published:** 2015-05-15

**Authors:** Ya-Lan Wang, Nasim Mohammadi Estakhri, Amber Johnson, Hai-Yang Li, Li-Xiang Xu, Zhenyu Zhang, Andrea Alù, Qu-Quan Wang, Chih-Kang (Ken) Shih

**Affiliations:** 1 Department of Physics, The University of Texas at Austin, Austin, TX 78712, USA; 2 Department of Physics, Wuhan University, Wuhan 430072, P. R. China; 3 Department of Electrical and Computer Engineering, The University of Texas at Austin, Austin, TX 78712, USA; 4 ICQD/HFNL, University of Science and Technology of China, Hefei 230026, P. R. China

## Abstract

By using silver nanoplatelets with a widely tunable localized surface plasmon resonance (LSPR), and their corresponding local field enhancement, here we show large manipulation of plasmonic enhanced upconversion in NaYF4:Yb^3+^/Er^3+^ nanocrystals at the single particle level. In particular, we show that when the plasmonic resonance of silver nanolplatelets is tuned to 656 nm, matching the emission wavelength, an upconversion enhancement factor ~5 is obtained. However, when the plasmonic resonance is tuned to 980 nm, matching the nanocrystal absorption wavelength, we achieve an enhancement factor of ~22 folds. The precise geometric arrangement between fluorescent nanoparticles and silver nanoplatelets allows us to make, for the first time, a comparative analysis between experimental results and numerical simulations, yielding a quantitative agreement at the single particle level. Such a comparison lays the foundations for a rational design of hybrid metal-fluorescent nanocrystals to harness the upconversion enhancement for biosensing and light harvesting applications.

The upconversion photoluminescence (PL), a process resulting in the emission of photons with higher energy than the excitation source^1^,^2^, has raised significant attention due to its potential applications in biomedical imaging^3^,^4^, solar cells^5^,^6^, solid state lasers^7^,^8^, and solid state lighting^9^,^10^. The upconversion fluorescence spectrum typically ranges from near-infrared (NIR) to visible, with different combinations of host and dopant. Rare-earth salts such as NaYF_4_ nanocrystals have been considered as promising hosts. They are often co-doped with Yb^3+^/Er^3+^ to obtain better emission efficiencies^11^, ^12^, ^13^, ^14^, ^15^, ^16^, ^17^. However, due to the small absorption cross section of rare-earth ions, the upconversion efficiency still remains quite limited.

Manipulating light-matter interactions at the nanoscale using metallic nanostructure has been shown to be a very promising route for enhancing light emitters via plasmonic resonances^18^,^19^,^20^,^21^,^22^,^23^,^24^,^25^,^26^,^27^,^28^,^29^,^30^,^31^,^32^,^33^,^34^,^35^,^36^,^37^,^38^,^39^,^40^,^41^,^42^,^43^,^44^,^45^. Examples include plasmonic enhanced spontaneous emission^25^,^26^,^27^,^28^,^29^,^30^,^31^,^32^, Raman scattering^33^,^34^,^35^,^36^,^37^,^38^,^39^,^40^,^41^, and nonlinear optical processes^42^,^43^,^44^. This strategy has also been adopted to enhance the upconversion efficiency for rare-earth salts^45^,^46^,^47^,^48^,^49^,^50^,^51^,^52^,^53^,^54^,^55^,^56^,^57^,^58^,^59^. There are two ways to use plasmonic resonances to achieve upconversion enhancement: matching the plasmonic resonance to the absorption wavelength (referred to as absorption matching), or matching it to the emission wavelength (referred to as emission matching). Both possibilities have been recently demonstrated by Kagan’s group in NaYF_4_ nanoparticles doped with lanthanide^47^,^48^. It was found that the absorption matching is a more efficient strategy, as expected since the rate limiting step of the up-conversion process is the two photon absorption. All studies thus far relied on ensemble measurements, not allowing a direct verification of the microscopic mechanisms behind this phenomenon. For example, is the upconversion enhancement simply due to resonance frequency matching, or does the field enhancement play an additional role? More importantly, can one quantitatively account for each factor?

In this communication, these critical questions are addressed here by manipulating the coupling of upconversion fluorescent nanoparticle (NaYF_4_:Yb^3+^/Er^3+^) to plasmonic nanostructures (silver nanoplatelets) at the single particle level. Experimentally, we track single fluorescent particles before and after coupling to triangularly shaped silver nanoplatelets (AgNPs), whose plasmon resonance is widely tunable by adjusting the lateral length. Significantly, we determine the actual structural position of single emitters and plasmonic enhancers, allowing a quantitative comparison between experimental results and numerical modeling ( http://www.CST.com) at the single particle level^43^. To our knowledge this is the first direct quantitative comparison of plasmonic enhanced upconversion at the single nanoparticle level between experiment and theoretical simulation. Thus, it will allow a reliable and rational design of plasmonic/fluorescent hybrid systems at the nanoscale for practical applications, as well as a better understanding of the microscopic phenomena at the basis of this process.

## Results

**Figure 1:**
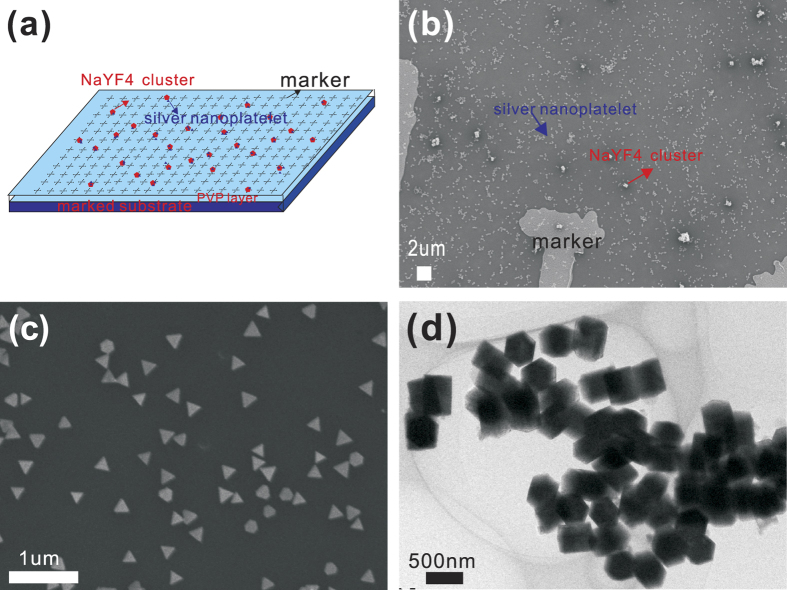
Schematic and morphology characterization of the samples. (**a**) The schematic of the three-layer sample deposited on the marked substrate; (**b**) SEM image of the structure; (**c**) SEM image of AgNPs resonating at 980 nm; (**d**) TEM image of the NaYF_4_:Yb^3+^/Er^3+^ nanocrystals doped with 10% Mn^2+^.

In order to track individual fluorescent nanoparticles before and after plasmonic enhancement, we created a substrate with registration markers serving as a constellation coordinate for single nanoparticles (see schematic shown in Fig. 1a). Figure 1b shows the corresponding scanning electron microscope (SEM) image for a typical sample, in which the registration markers can be clearly identified. In this image, the NaYF_4_:Yb^3+^/Er^3+^ nanocrystals and the triangular AgNPs can also be seen. The sample was fabricated with a three-step process: first, the upconversion nanocrystals, originally in ethanol solution with a concentration of 1 mg/ml, were spun on the marked silicon substrate with native oxide. Then, a thin layer of polyvinyl pyrrolidone (PVP) was spun on top of the emitter layer to control the space between the emitter layer and the AgNP layer (see more details in Supporting Information). We measured the fluorescence of the emitters before depositing AgNPs as plasmonic enhancers and then tracked them again after the deposition of the AgNPs as the third layer using spin coating.

AgNPs were synthesized following a method previously reported^52^, based on which various sizes and thicknesses of AgNPs can be easily obtained by regulating the ratio of the silver nitrate and sodium citrate and other chemicals in the synthesis process. The size of the AgNPs can also be easily controlled, since it linearly grows with the synthesis time. As an example, the AgNPs shown in Fig. 1c have a triangular shape with a uniform edge length of ~170 nm and a thickness of ~11 nm, tuned to resonate at 980 nm.

**Figure 2:**
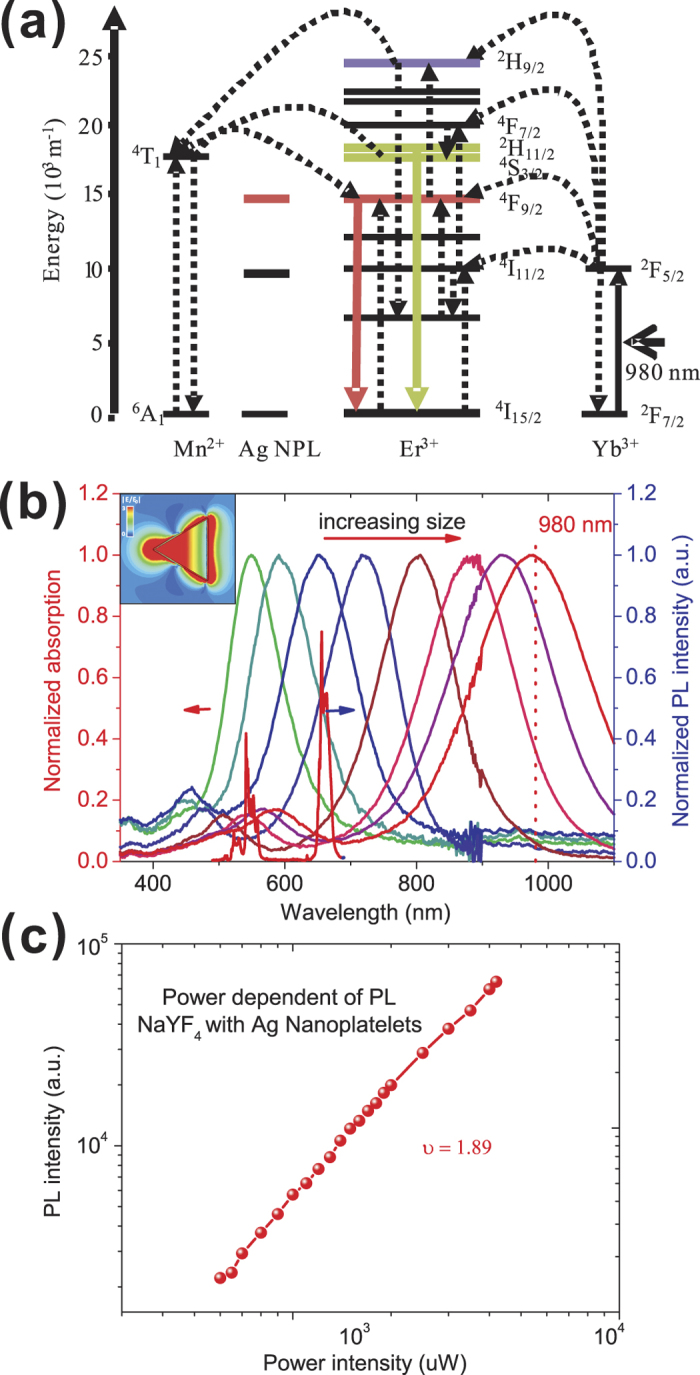
The energy diagram and power-dependent emission spectra of the sample. (**a**) Detailed energy diagram of the NaYF_4_:Yb^3+^/Er^3+^ doped with Mn^2+^, which describe the upconversion processes under analysis here; (**b**) Dependence of the local surface plasmon resonance on the size of the AgNPs and PL spectra of the doped NaYF_4_ nanocrystals (blue line); the inset shows the field distribution of single resonant AgNP at 980 nm; (**c**) Log-log plot of the light emission intensity as a function of excitation power.

In Fig. 1d we show the synthesized NaYF_4_:Yb^3+^/Er^3+^ nanocrystals doped with Mn^2+^, with a uniform diameter of ~300 nm. The upconversion mechanism in Yb^3+^/Er^3+^ systems has been discussed extensively before^17^. The absorption is mediated by the ^2^F_7/2_ to ^2^F_5/2_ transition in Yb^3+^. The higher level excitations in Er^3+^ ions occur via several different channels of energy transfer, as sketched in Fig. 2a. There are two main emission bands (labeled by the green and red arrows in the figure) centered at 530–560 nm and 640–670 nm, ascribing to the radiative energy transfer from ^4^S_3/2_ to ^4^I_15/2_ and ^4^F_9/2_ to ^4^I_15/2_ level of Er^3+^, respectively. The resulting upconversion emission spectrum is shown in Fig. 2b, labeled by the red line. Also shown in Fig. 2c is the log-log plot of the light emission intensity as a function of excitation power. A slope of ~2 (measured 1.89) in the log-log plot confirms that the rate limiting step is the two-photon absorption process.

The most attractive aspect of the proposed AgNPs as a platform to enhance the upconversion process is their widely tunable plasmonic resonance. Shown in Fig. 2b are plasmonic resonances of AgNPs with different lateral sizes, spanning from green to NIR wavelengths. This tunability allows us to realize “emission matching” or “absorption matching” by simply using different lateral sizes of AgNPs, still keeping their size overall subwavelength. Also shown in the inset is the field distribution of the resonantly-excited single AgNP at the sample wavelength of 980 nm.

**Figure 3:**
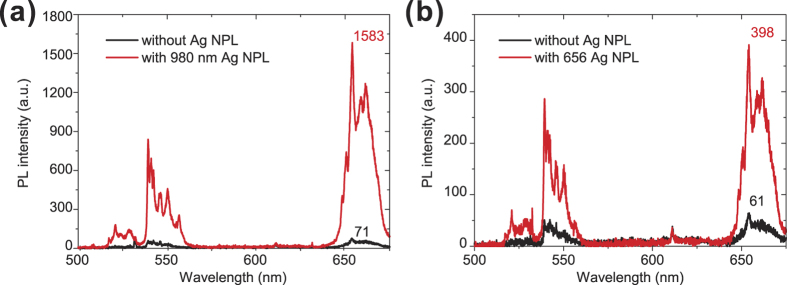
PL upconversion spectra of the sample. PL upconversion spectra of single nanocrystals with and without AgNPs, when the LSPR is tuned to resonate at (**a**) 980 nm and (**b**) 656 nm.

Figure 3a shows the upconverted luminescent spectra of a single nanoscrystal with (red line) and without (black line) AgNPs, with plasmonic resonance matching the absorption line (980 nm). For this nanocrystal, we observe a 22-fold enhancement of PL due to the local field enhancement by the AgNPs. This enhancement is different among emitters, since it is at the single emitter level (as shown in Supporting Information **Fig. S2**). The enhancement factor is wavelength-dependence, as studied in the Ref. 48 and also displayed in the Supporting Information **Fig. S3**. In contrast, when “emission matching” AgNPs are used, only an enhancement factor of 5 is achieved (Fig. 3b). This comparison upholds the notion that absorption matching is more efficient as reported earlier.

**Figure 4:**
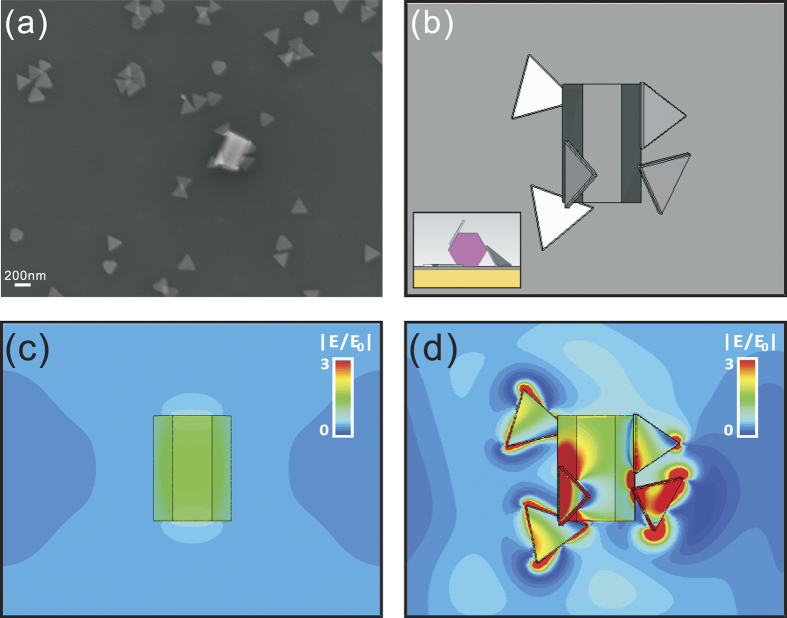
The simulation results of the sample. (**a**) SEM image of the composite system, one upconversion nanocrystal surrounded by five AgNPs; (**b**) The 3D structure used in the simulation, top and cross view; Field distributions around the emitter (**c**) in the absence and (**d**) in the presence of the AgNPs.

To gain further insight into the phenomena underlying this large upconversion enhancement, we numerically simulated the field enhancement for the upconversion nanocrystal surrounded by AgNPs. The AgNPs used in this work have low losses and allow suppressing scattering, this has been confirmed by our before simulation works on the plasmonic metal-plate cloaks^60^,^61^. Figure 4a shows the actual geometry of the configuration (SEM image), where a single upconversion nanocrystal is surrounded by multiple AgNPs, and Fig. 4b shows the corresponding three-dimensional simulation setup (top view and side view), matching the experimental image.

The field distribution around the emitter with and without AgNPs is shown in Fig. 4c, 4d, respectively. Based on the simulation of the local field enhancement, and the integration over the emitter, we obtain a factor of 27 for the total enhancement of absorption, which is quantitatively consistent with the experimental result of ~22. From the theoretical standpoint, the enhancement in radiated power is proportional to the fourth power of the near field enhancement at the absorption wavelength, as the upconversion is a two photon process^62^. As a result, the resonant excitation of nanoparticles is theoretically predicted to create the strongest enhancement of emission due to the large near-field enhancement in the proximity of resonant NPs (inset of Fig. 2b and Fig. 4d). On the other hand, NPs resonant at the emission wavelength contribute to upconversion improvement through the enhancement of the density of states at the location of the emitters, which is a linear process, and at the same time increase the radiative emission rate^13^,^47^, resulting in an smaller overall effect.

We emphasize that the good quantitative comparison between theory and experiment is made possible because we can track individual emitters before and after coupling to the AgNPs, and obtain the actual geometric relation between the emitter and the plasmonic enhancers at the single emitter level. The quantitative agreement between theoretical simulation and the experimental results further illustrates that a microscopic, quantitative description of plasmonic enhanced upconversion can fully account for this phenomenon.

In conclusion, we have investigated the upconversion enhancement of NaYF_4_:Yb^3+^/Er^3+^ nanocrystals at the single particle limit, based on LSPR supported by AgNPs. We experimentally obtain an enhancement of over 22 folds when the LSPR of AgNPs resonates with the excitation wavelength, consistent with our numerical calculations and theoretical modeling. Optimal setups may be envisioned in which both sets of nanoparticles are carefully placed to enhance both absorption and emission wavelengths. We also envision that with lithographic tools it may be possible to largely improve the positioning and control of the AgNPs, in order to further boost the overall upconversion efficiency. This strategy for PL enhancement provides exciting venues for applications in biosensing and solar cell technology

## Methods

### Preparation of AgNPs and upconversion nanocrystals

Seed-mediated growth method was applied to synthesize the silver nanoplatelets. Two steps were involved. In the first step, the suspension of seeds were prepared as follows: 147 ml DI water was mixed with 9 ml of sodium citrate (Na_3_CA) (30 mmol/ml) under magnetic stirring. Then, 9 ml of polyvinyl pyrrolidone (PVP) (MW ~ 40000, 20.3 mg/ml), 0.36 ml of hydrogen peroxide (H_2_O_2_) (30%), 3 ml of silver nitrate (AgNO_3_) (0.85 mg/ml), and 1.5 ml of sodium borohydride (NaBH_4_) (3.78 mg/ml) were added into the solution in sequence. The second step starts by placing 10 ml of as-prepared silver nanoplatelet seeds into a flask. Then 10 ml of aqueous solution containing L-ascorbic acid (AA) (1.2 mmol/ml) and Na_3_CA (0.4 mmol/ml) were insert into the seeds under magnetic stirring. It was then followed by the injection of AgNO_3_ solution (0.6 mmol/ml) using a syringe pump at a rate of 10 mL/h. The silver nanoplatelets with different plasmonic resonance wavelength were pulled out at different time and collected by centrifugation at 11000 rpm for 12 minutes with water tow times under 4 degrees.

Upconversion nanocrystals doped with Mn^2+^ were synthesized using a typical procedure. After synthesized and naturally cooling, the sample was centrifuged by ethanol and water for four times, dried in a vacuum tank for 12 h at 65 °C for further usage.

### Preparation of Three-Layer Thin Film

AgNPs were synthesized using the method of Jie Zeng with a little modification. After synthesis, they were centrifuged and re-dispersed into ethanol at a speed rate of 12000 r/min for 12 minutes under 4 ^o^C. PVP solutions were directly dissolved in the ethanol. Upconversion nanocrystals were re-dispersed into the ethanol with the concentration of 1 mg/ml. All solutions were transferred to the films on the substrate using the spin-coating method using a spinner (spin150) at a speed of 4000 r/min. The marked substrates were made by photolithography method.

### Optical Measurements

The samples used for transmission electron microscope (TEM) characterization were dropped on copper grids and dried at room temperature. TEM images were taken on JEOL 2010 FET transmission electron microscope (operated at 200 kV). The AFM images were taken on Multimode scanning probe microscope (MM-SPM).

The upconversion photoluminescence (PL) were collected by the reflection measurement. An *p*-polarized laser for the measurements of PL was generated by a pulsed Ti:Sapphire laser with a pulse width ~3 ps and a repetition rate 76 MHz. The excitation wavelength was tuned to 980 nm. The PL from the sample was collected by anx100 objective. The PL spectra were recorded by using a spectrometer (Spectrapro 2500i, Acton) coupled with a liquid nitrogen cooled CCD.

## Author Contributions

The samples of silver nanoplates and upconversion nanocrystals were prepared by Y.L.W., H.Y.L.; the experimental measurements and data collection were carried out by Y.L.W., A.J., and L.X.X. with assistance of C.K.S. and A.A.; data analysis and theoretical modeling were performed by Y.L.W. N.M.E., A.A. and C.K.S. The manuscript was written by A.A., C.K.S. and Q.Q.W. with assistance of Y.L.W. and Z.Z.; the project was supervised by Z.Z., Q.Q.W., A.A. and C.K.S.

## Additional Information

**How to cite this article**: Wang, Y.-L. *et al*. Tailoring Plasmonic Enhanced Upconversion in Single NaYF_4_:Yb^3+^/Er^3+^ Nanocrystals. *Sci. Rep.* **5**, 10196; doi: 10.1038/srep10196 (2015).

## Supplementary Material

Supporting Information
