# Supplementary material for: Tailoring Plasmonic Enhanced Upconversion in Single NaYF4:Yb3+/Er3+ Nanocrystals
Source: Sci Rep. 2015 May 15;5:10196. doi: 10.1038/srep10196 (PMC4432370; doi:10.1038/srep10196)
Supplement: Supporting Information [file srep10196-s1.doc]

Supplementary Information

**“****Tailoring Plasmonic Enhanced Upconversion in Single NaYF_4_:Yb^3+^/Er^3+^ Nanocrystals”** *by Ya-Lan Wang, Nasim Mohammadi Estakhri, Amber Johnson, Hai-Yang Li, Li-Xiang Xu, Zhenyu Zhang, Andrea Alu, Qu-Quan Wang, Chih-Kang (Ken) Shih*

**1. Synthesis procedure and characterize methods**

Seed-mediated growth method was applied to synthesize the silver nanoplatelets. Two steps were involved. In the first step, the suspension of seeds were prepared as follows: 147 ml DI water was mixed with 9 ml of sodium citrate (Na_3_CA) (30 mmol/ml) under magnetic stirring. Then, 9 ml of polyvinyl pyrrolidone (PVP) (MW ~ 40000, 20.3 mg/ml), 0.36 ml of hydrogen peroxide (H2O2) (30%), 3 ml of silver nitrate (AgNO_3_) (0.85 mg/ml), and 1.5 ml of sodium borohydride (NaBH_4_) (3.78 mg/ml) were added into the solution in sequence. The second step starts by placing 10 ml of as-prepared silver nanoplatelet seeds into a flask. Then 10 ml of aqueous solution containing L-ascorbic acid (AA) (1.2 mmol/ml) and Na_3_CA (0.4 mmol/ml) were insert into the seeds under magnetic stirring. It was then followed by the injection of AgNO_3_ solution (0.6 mmol/ml) using a syringe pump at a rate of 10 mL/h. The silver nanoplatelets with different plasmonic resonance wavelength were pulled out at different time and collected by centrifugation at 11000 rpm for 12 min with water tow times under 4 degree.

Upconversion nanocrystals doped with Mn^2+^ were synthesized using a typical procedure. After synthesized and naturally cooling, the sample was centrifuged by ethanol and water for four times, dried in a vacuum tank for 12 h at 65 °C for further usage. AgNPs were synthesized using the method of Jie Zeng with a little modification. After synthesis, they were centrifuged and re-dispersed into ethanol at a speed rate of 12000 r/min for 12 minutes under 4^o^C. PVP solutions were directly dissolved in the ethanol. Upconversion nanocrystals were re-dispersed into the ethanol with the concentration of 1 mg/ml. All solutions were transferred to the films on the substrate using the spin-coating method using a spinner (spin150) at a speed of 4000 r/min. The marked substrates were made by photolithography method.

The samples used for transmission electron microscope (TEM) characterization were dropped on copper grids and dried at room temperature. TEM images were taken on JEOL 2010 FET transmission electron microscope (operated at 200 kV). The AFM images were taken on Multimode scanning probe microscope (MM-SPM).

The upconversion photoluminescence (PL) were collected by the reflection measurement. An *p*-polarized laser for the measurements of PL was generated by a pulsed Ti:Sapphire laser with a pulse width ~ 3 ps and a repetition rate 76 MHz. The excitation wavelength was tuned to 980 nm. The PL from the sample was collected by anx100 objective. The PL spectra were recorded by using a spectrometer (Spectrapro 2500i, Acton) coupled with a liquid nitrogen cooled CCD.

**2. Power-dependent two-photon upconversion spectrum of the NaYF4:Yb^3+^/Er^3+^ doped with Mn^2+^**

**Figure S1** shows the PL spectra of the sample as a function of the excitation power intensity.


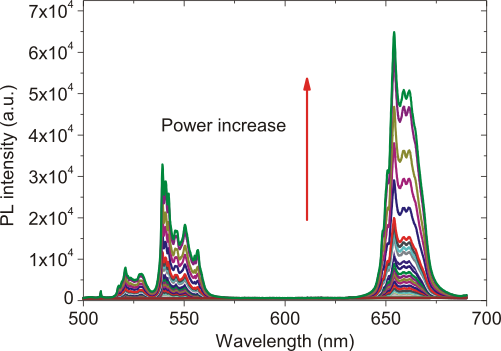


Figure S1. PL spectra of the sample as a function of the excitation power intensity.

**3. The upconversion spectra of different NaYF4:Yb^3+^/Er^3+^ nanocrystals doped with Mn^2+^ at single nanoparticle level.**

**Figure S2** shows the SEM images and PL spectra of different single NaYF4:Yb^3+^/Er^3+^ nanocrystals doped with Mn^2+^. We can see the enhancement ratio slightly varies with the position and number of Ag nanoplatelets.


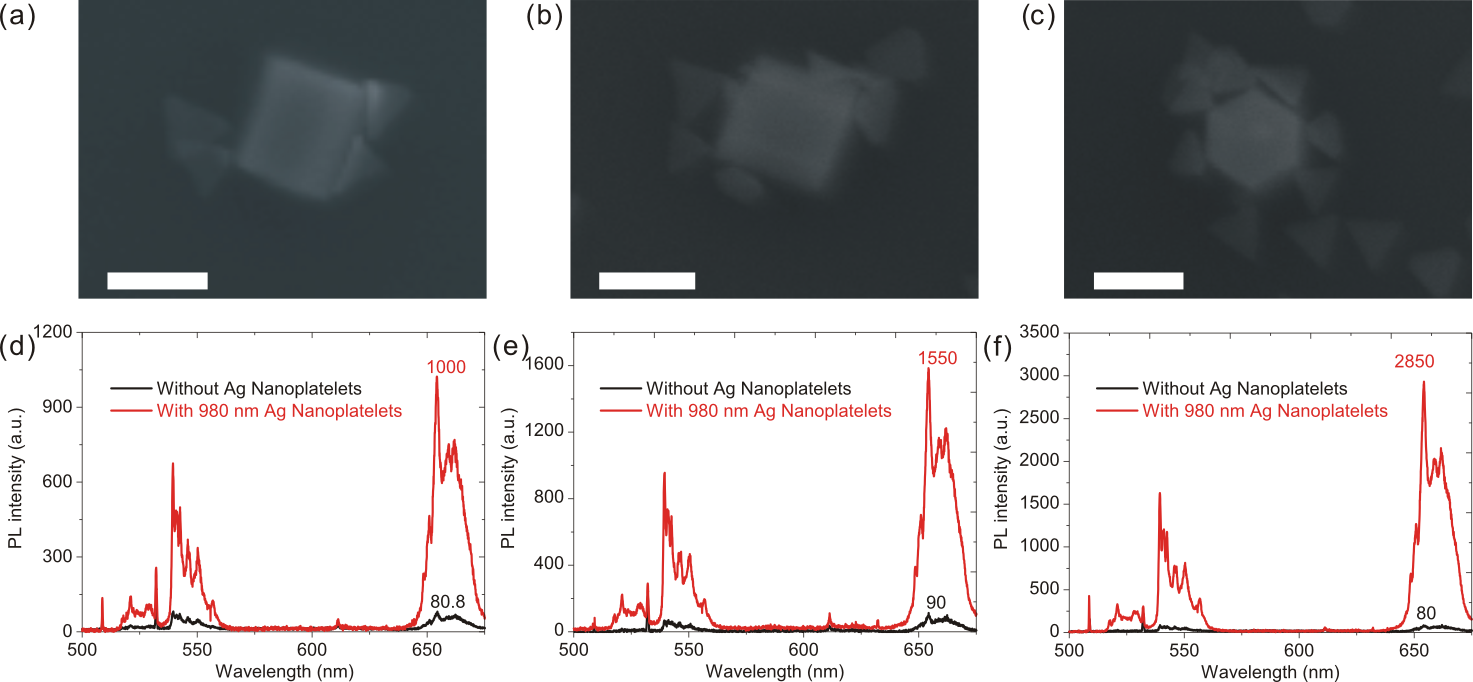


Figure S2. SEM images (bar is 200 nm) and PL spectra of different single NaYF4:Yb^3+^/Er^3+^ nanocrystals doped with Mn^2+^.

**4. The wavelength dependence of the upconversion emission enhancement of the NaYF4:Yb^3+^/Er^3+^ nanocrystals doped with Mn^2+^ by the Ag nanoplates.**

**Figure S3** shows the wavelength dependence of the upconversion emission enhancement of the NaYF4:Yb^3+^/Er^3+^ nanocrystals doped with Mn^2+^ by the Ag nanoplates with LSPR ~650 nm and ~550 nm, respectively.


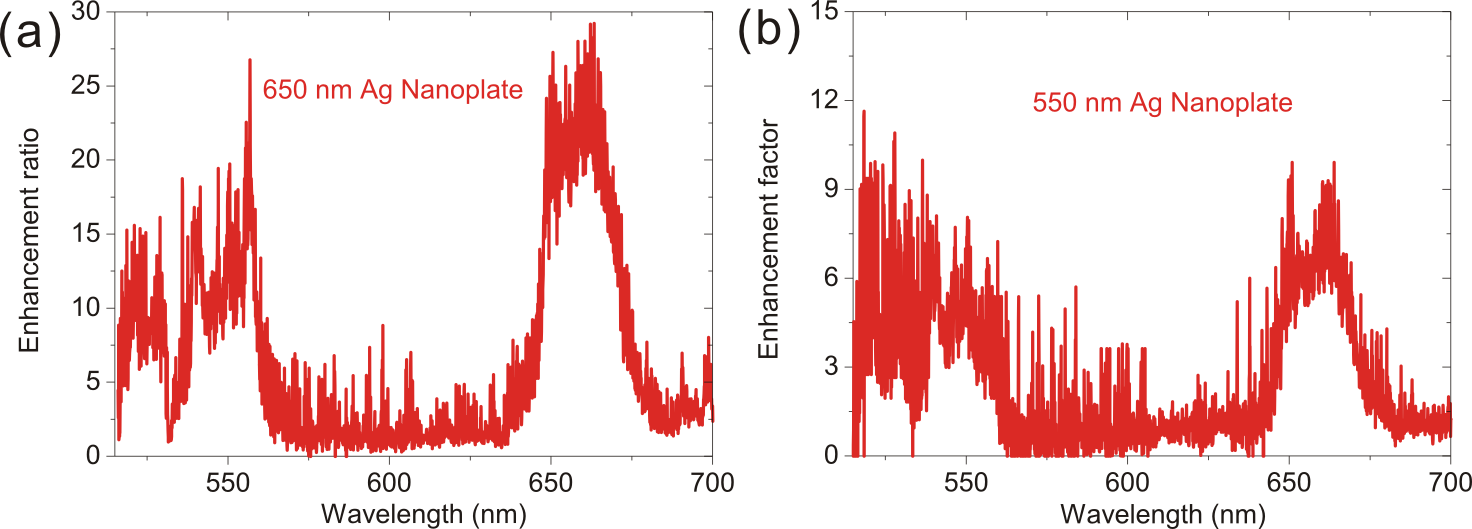


Figure S3. the wavelength dependence of the upconversion emission enhancement of the NaYF4:Yb^3+^/Er^3+^ nanocrystals doped with Mn^2+^ by the Ag nanoplates with LSPR ~650 nm (a) and ~550 nm (b), respectively.

**5. Temperature-dependent upconversion spectra of the NaYF4:Yb^3+^/Er^3+^ doped with Mn^2+^.**

**Figure S4** presents the temperature dependent upconversion spectra of the NaYF_4_:Yb^3+^/Er^3+^ doped with Mn^2+^. The 40 degrees will have ~20 % decrease compared to the room temperature (19 degrees).


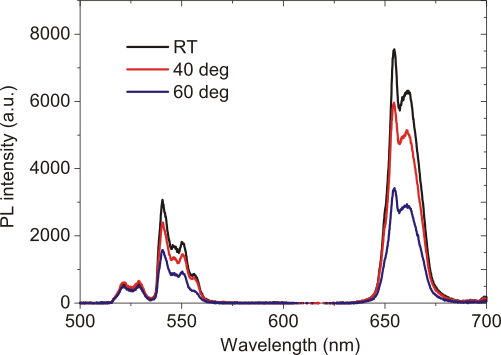


Figure S4. the temperature dependent upconversion spectra of the NaYF_4_:Yb^3+^/Er^3+^ doped with Mn^2+^.

**6. The upconversion spectra of the NaYF4:Yb^3+^/Er^3+^ doped with and without Mn^2+^.**

**Figure S5** presents the upconversion spectra of the NaYF_4_:Yb^3+^/Er^3+^ doped with and without Mn^2+^. The doping of Mn^2+^ leads to ~10 folds of enhancement of emission intensity.


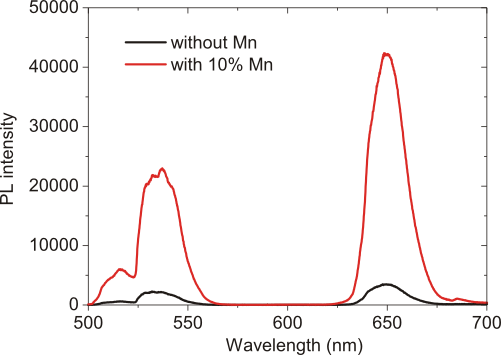


Figure S5 presents the upconversion spectra of the NaYF_4_:Yb^3+^/Er^3+^ doped with and without Mn^2+^.
